# Integrin Beta 1 Is Crucial for Urinary Concentrating Ability and Renal Medulla Architecture in Adult Mice

**DOI:** 10.3389/fphys.2018.01273

**Published:** 2018-09-13

**Authors:** Anna Iervolino, Luigi R. De La Motte, Federica Petrillo, Federica Prosperi, Francesca Maria Alvino, Guglielmo Schiano, Alessandra F. Perna, Danilo Di Matteo, Mario De Felice, Giovambattista Capasso, Francesco Trepiccione

**Affiliations:** ^1^Biogem Scarl, Istituto di Ricerche Gaetano Salvatore, Ariano Irpino, Italy; ^2^Department of Translational Medical Sciences, University of Campania “Luigi Vanvitelli”, Naples, Italy; ^3^Department of Molecular Medicine and Medical Biotechnologies, University of Naples “Federico II”, Naples, Italy

**Keywords:** integrin beta1, collecting duct, thick ascending limb, renal failure, AQP2

## Abstract

Integrins are heterodimers anchoring cells to the surrounding extracellular matrix (ECM), an active and complex process mediating a series of inside-out and outside-in stimuli regulating cellular turn-over, tissue growth and architecture. Itgb1 is the main subunit of the renal integrins and it is critical for renal development. This study aims to investigate the role of Itgb1 in the adult renal epithelial cells by knocking down Itgb1 in PAX8 expressing cells. *Itgb1*-*Pax8* cKO mice develop a progressively worsening proteinuria and renal abnormalities leading to severe renal failure and hypertension. This phenotype is also associated with severe dysfunction of distal nephron and polyuria. To further investigate whether distal nephron involvement was primarily related to Itgb1 suppression or secondary to renal failure, an *Itgb1*-*AQP2* cKO mouse model was generated. These mice lack Itgb1 expression in AQP2 expressing cells. They do not show any developmental alteration, but 1 month old mice are resistant to dDAVP administration and finally, at 2 months of age, they develop overt polyuria. This phenotype is due to primary collecting duct (CD) cells *anoikis*. The entire architecture of the outer medulla is altered, with loss of the typical organization pattern of vascular and tubular bundles alternation. Indeed, even though not primarily affected by genetic ablation, the TAL is secondarily affected in this model. It is sufficient to suppress Itgb1 expression in the CD in order to stimulate proliferation and then disappearance of neighboring TAL cells. This study shows that cell to cell interaction through the ECM is critical for architecture and function maintenance of the outer medulla and that Itgb1 is crucial for this process.

## Introduction

Integrins are transmembrane heteromeric receptors that mediate the interactions between cells and extracellular matrix (ECM). They consist of non-covalently bound α and β subunits combining in a restricted manner to form specific αβ dimers. In mammals, the 18α and 8β subunits form more than 20 different dimers, each of which exhibiting different ligand binding properties (Hynes, 2002; Legate et al., 2009). Integrins α3β1, α6β1, and α6β4 are the main laminin binding receptors, while integrins α1β1 and α2β1 are the predominant collagen receptors. Although primarily thought as anchoring molecules, integrins play a crucial role in cell adhesion, migration, proliferation and apoptosis by transducing signals through their cytoplasmic tails following ligand binding (Ginsberg et al., 2005; Legate and Fassler, 2009; Moser et al., 2009).

Itgb1 is the most abundantly expressed β subunit and is found in almost all cell types in the body, including the kidney where it is highly expressed in the glomerulus and tubules (Kreidberg and Symons, 2000).

Integrins are crucial for kidney development. Mice carrying mutations in Itga3 showed severe renal and lung development alterations causing premature death at neonatal age. Constitutive deletion of integrins results in defective gastrulation and embryos death (Stephens et al., 1995). This is the main reason why conditional transgenic models have been extensively used to address tissue specific function of integrin subunits. In this regard, Itgb1 has been shown to be important for collecting duct (CD) morphogenesis. In fact, if deleted at E9.5, when the ureteric bud morphogenesis starts, Itgb1 suppression severely affects CD branching and leads to a wide range of renal gross morphology abnormalities (Wu et al., 2009; Zhang et al., 2009). Indeed, HoxB7-Itgb1 cKO mice present with single or double kidney agenesis or inner medulla hypoplasia. *In vitro* evidences support also a direct role of Itgb1 for AQP2 function, particularly for its sorting to the membrane (Tamma et al., 2011; Chen et al., 2012). Thus, Itgb1 seems to be particularly crucial either for CD structure or function during development relatively to adult mice.

Recent studies suppressing Itgb1 in cells expressing AQP2 (E15.5–E17.5) showed different results likely related to different recombination efficiency of the transgenic model used. Zhang et al. (2009) showed a mild phenotype of CDs dysfunction secondary to Itgb1 ablation, while Mamuya et al. (2017) report a model of severe urinary concentrating defect.

To investigate the role of Itgb1 beyond renal development, a cKO model specific in renal epithelial cells and induced later in nephrogenesis has been generated. This model results in severe renal failure by targeting mainly the function of the glomerulus and distal nephron. To further dissect Itgb1 role in distal nephron function we selectively knocked down Itgb1 in AQP2 expressing cells. With this approach we show here that Itgb1 is cornerstone for medullary architecture maintenance and urinary concentrating ability in adult mice and the selective ablation of Itgb1 in the CD is sufficient to affect the TAL as well.

## Results

### Ablation of Itgb1 in Renal Epithelial Cells Induces Severe Impairment of Renal Function

To address the role of Itgb1 in adult renal epithelial cells we generated a transgenic mouse model lacking the Itgb1 gene in Pax8 expressing cells, namely *Itgb1*^f/f^-*Pax8*^cre/+^ mice.

The beta-galactosidase activity was a marker of efficient CRE-LOX recombination. Indeed, LoxP-flanked *Itgb1* gene is carrying a promoterless *lacZ* reporter gene. When and where the CRE recombines with the floxed DNA region of the Itgb1 gene, it promotes the expression of *lacZ* together with the excision of the Itgb1 region of interest. Following this approach, exposing tissue beta-galactosidase to X-GAL, a blue staining develops in the cells that have efficiently undergone CRE-LOX recombination.

**FIGURE 1:**
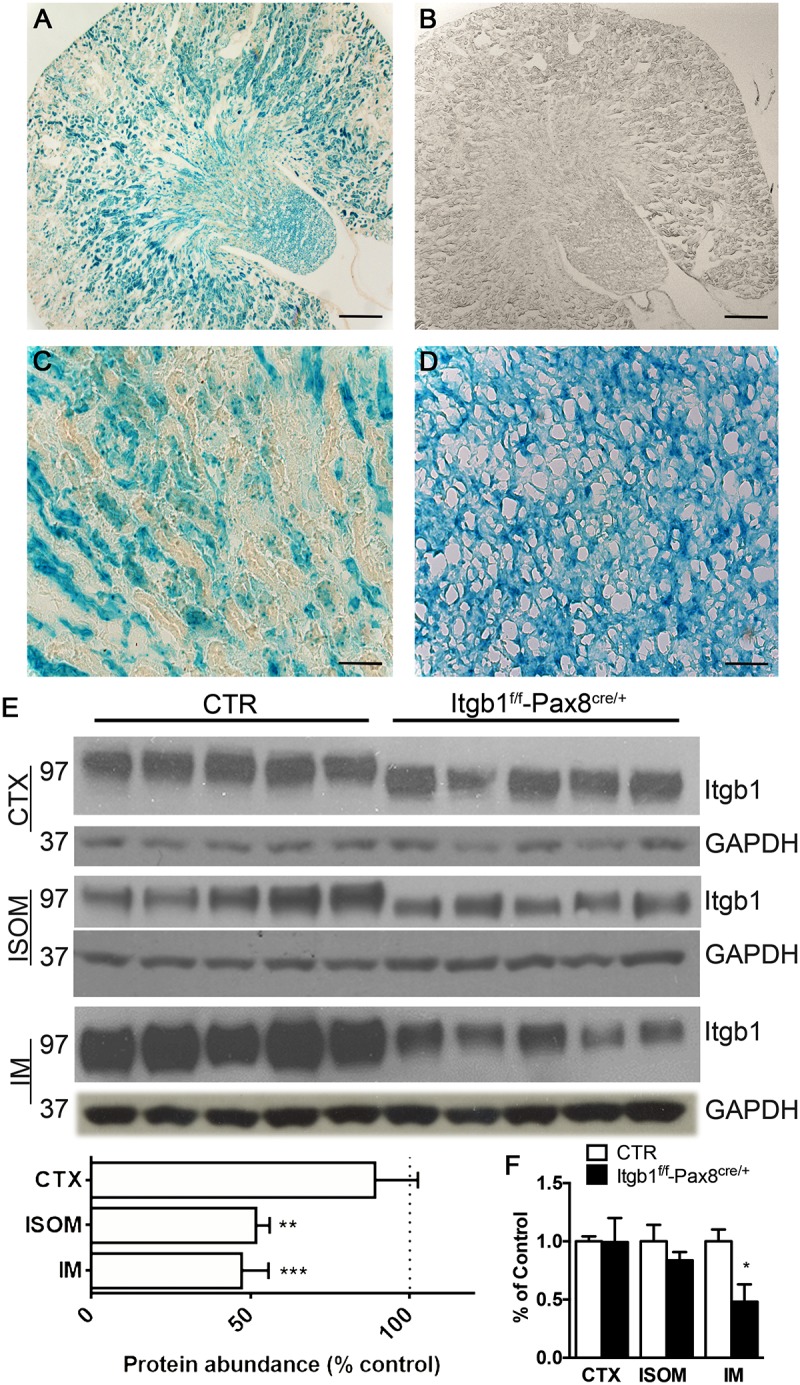
Model validation Itgb1^f/f^-Pax8^cre/+^. **(A–D)** Show the beta-galactosidase assay. In Itgb1^f/f^-Pax8^cre/+^ **(A)** a blue staining indicates the presence of beta galactosidase, as effect of CRE-LOX recombination, in the whole kidney, but not in the control Itgb1^f/f^ **(B)** (scale bar 250 μm). At higher magnification the vast majority of cortical **(C)** and medullary **(D)** cells underwent to an efficient recombination (scale bar 20 μm). **(E)** Shows by immunoblotting, the significant downregulation of Itgb1 in Itgb1^f/f^-Pax8^cre/+^ compared to control Itgb1^f/f^ Data are expressed as mean ± sem; n power is 5 vs. 5, ^∗^*p* < 0.05, ^∗∗^*p* < 0.01, and ^∗∗∗^*p* < 0.001 (unpaired *t*-test). **(F)** Shows data from mRNA quantification by qPCR both in the CTX, ISOM and IM. Data are expressed as mean ± sem; n power is 5 vs. 5, ^∗^*p* < 0.05 (unpaired *t*-test).

In **Figure 1**, the vast majority of renal epithelial cells both in the cortex and medulla showed efficient recombination (**Figures 1A,C,D**), while no staining is detected in control mice (**Figure 1B**). To corroborate these data, Itgb1 protein abundance was decreased throughout the kidney zones, mainly in the outer and inner medulla and slightly in the cortex of *Itgb1*^f/f^-*Pax8*^cre/+^ mice (**Figure 1E**). Expression level of Itgb1 mRNA recapitulates protein abundance in the cortex and inner medulla, a tendency toward downregulation was detected in the ISOM (**Figure 1F**).

**FIGURE 2:**
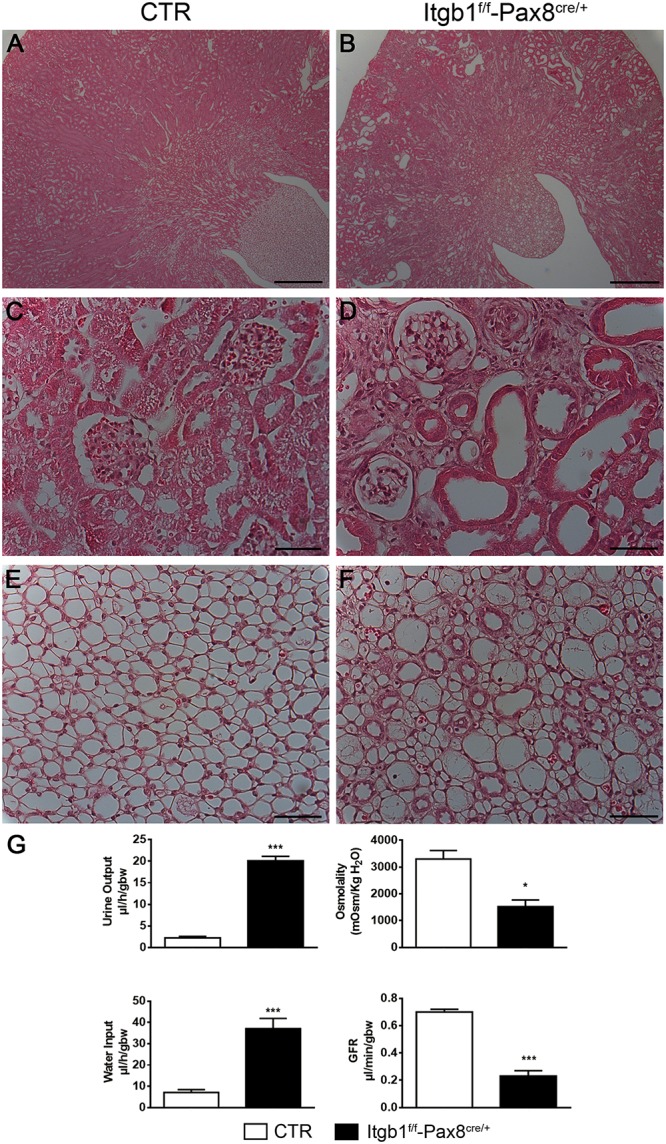
Itgb1^f/f^-Pax8^cre/+^ develop polyuria and tubular dilatation. Representative pictures of hematoxylin and eosin of 3 months old mice **(A–F)** show severe tubular dilatation of different segments of the nephron **(B)** both in the cortex **(D)** and medulla **(F)** of Itgb1^f/f^-Pax8^cre/+^ mice (scale bar 250 μm **A,B**; scale bar 20 μm **C–F**). **(G)** Shows physiological parameters from which 3 months old Itgb1^f/f^-Pax8^cre/+^ mice result to be affected by hyposmolar polyuria associated to increased water intake and signs of severe renal failure, as showed by lower inulin clearance. Data are expressed as mean ± sem, ^∗^*p* < 0.05 and ^∗∗∗^*p* < 0.001.

*Itgb1*^f/f^-*Pax8*^cre/+^ mice were born at Mendelian ratio and do not exhibit any gross kidney morphologic abnormalities compared with control. However, 3 months old *Itgb1*^f/f^-*Pax8*^cre/+^ mice presented a lower body weight (**Supplementary Table S1**) and the kidneys appear paler and with a rough shape compared with controls. At the same time point, microscopic examination shows tubular dilation, cysts and signs of hydronephrosis (**Figures 2A–F**).

These morphological alterations correlate with the development of hyposmotic polyuria and polydipsia starting at 1 month of age. Such alterations become massive at 3 months (**Figure 2G**). Severe proteinuria paralleled the urinary concentrating defect (**Supplementary Table S1**) and contributed to GFR decline as measured by FITC-inulin clearance (**Figure 2G**) and, finally, it leads to hypertension (**Supplementary Table S1**).

**FIGURE 3:**
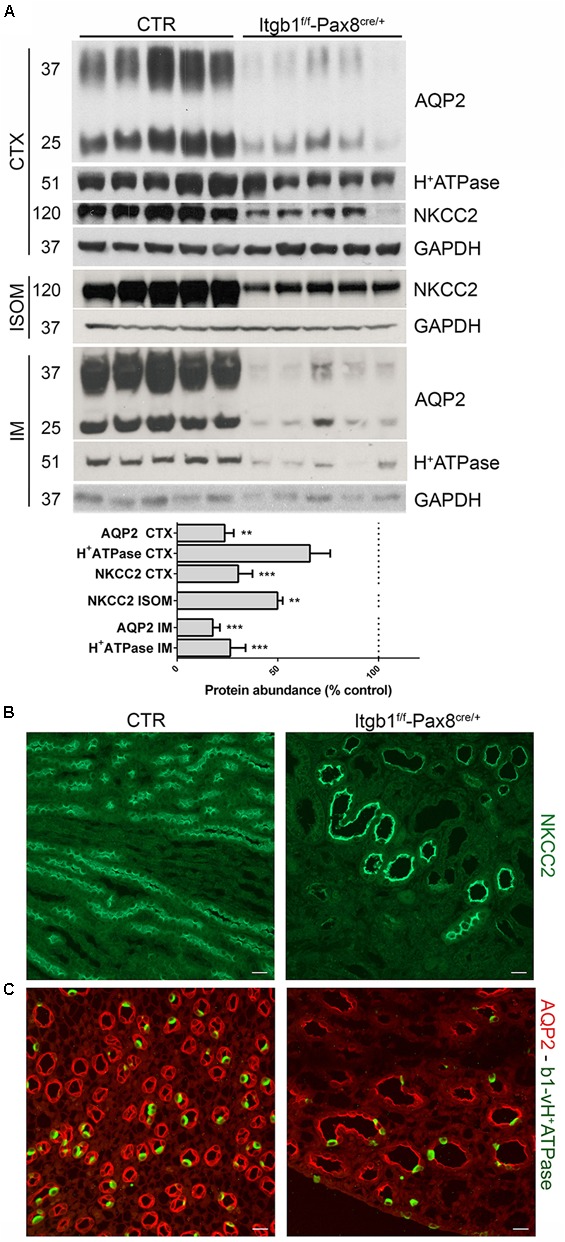
The polyuria in Itgb1^f/f^-Pax8^cre/+^ is associated to impaired function of TAL and CD. In **(A)** immunoblotting shows a severe downregulation of NKCC2, AQP2, and B1 subunit of vH^+^ATPase in 3 months old Itgb1^f/f^-Pax8^cre/+^ mice (data are expressed as mean ± sem; n power is 5 vs. 5. ^∗∗^*p* < 0.01 and ^∗∗∗^*p* < 0.001, unpaired *t*-test). In **(B)** representative pictures from ISOM stained with an anti-NKCC2 antibody, corroborates the fact that downregulation of NKCC2 in 3 months old Itgb1^f/f^-Pax8^cre/+^ mice is secondary to loss of TAL. In **(C)** representative pictures of IM stained with anti-AQP2 (red) and anti-B1-vH^+^ATPase (green) antibodies. At 3 months, Itgb1^f/f^-Pax8^cre/+^ mice showed a reduction in principal (AQP2+) and intercalated cells (B1-H^+^ATPase +) and a lower density of collecting ducts. Residual tubules show severe dilatation.

In order to evaluate the molecular determinants of the urinary concentrating defect, we analyzed the expression profile of proteins mainly involved in water transport. As showed in **Figure 3A**, Itgb1 suppression leads to severe downregulation of NKCC2 in ISOM and AQP2 and in the cortex and IM, suggesting that Itgb1 is required for both TAL and CD functions. These data were confirmed by immunofluorescence where no signs of intracellular retention or myslocalization of NKCC2 were detected (**Figure 3B**). In **Figure 3C**, double staining for AQP2 and B1 subunit of vH^+^-ATPase in 3 months old *Itgb1*^f/f^-*Pax8*^cre/+^ mice confirms that dysregulation of these proteins is due to a low density of CD and TAL in the inner medulla and in ISOM, respectively.

### Selective Suppression of Itgb1 in AQP2 Expressing Cells Causes Hyposmotic Polyuria Resistant to dDAVP in Adult Mice

Suppression of Itgb1 in Pax8 expressing cells impairs glomerular and tubular function leading to progressive renal failure. In this setting, the specific role of Itgb1 in water reabsorption homeostasis can be only inferred since the progressive development of chronic renal failure could impair water reabsorption along the distal nephron (Gong et al., 2003).

To further investigate this we generated a *Itgb1*^f/f^-AQP2^cre/+^ mice model.

**FIGURE 4:**
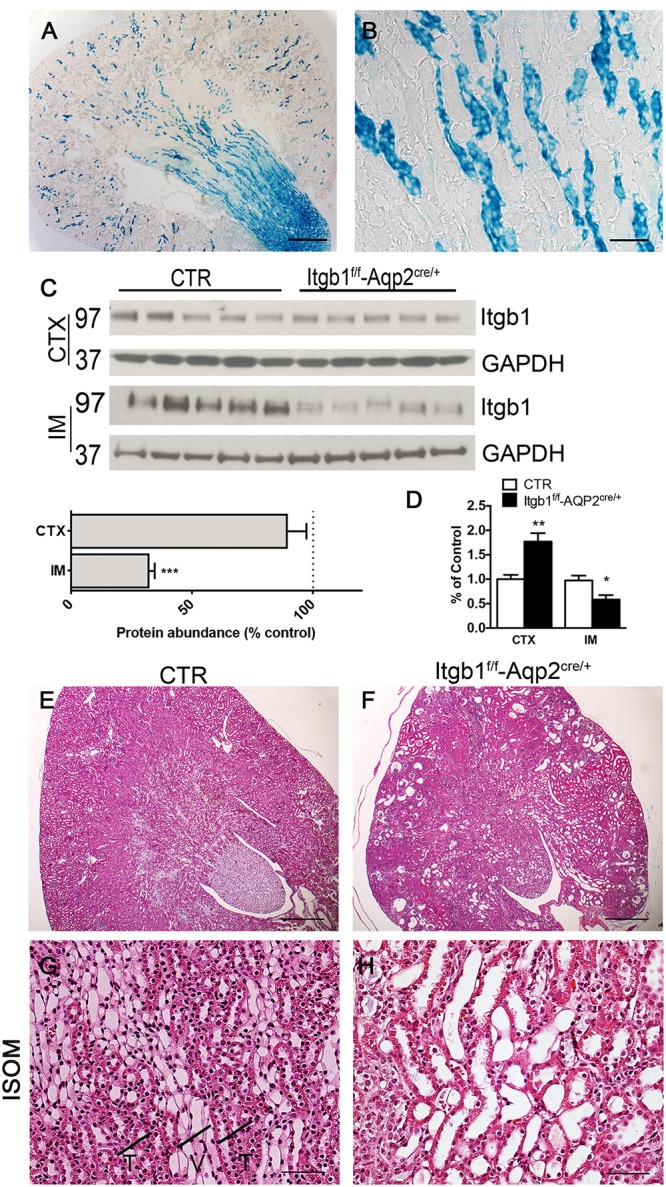
Model validation and histology of Itgb1^f/f^-Aqp2^cre/+^mice. **(A,B)** show the beta-galactosidase assay. The blue staining, labeling cells expressing beta galactosidase is limited to medullary rays of Itgb1^f/f^-Aqp2^cre/+^ mice **(A)** (scale bar 250 μm). At higher magnification from the ISOM **(B)**, only collecting ducts of the ISOM and not TAL underwent CRE-LOX recombination as expected by the transgene strategy (scale bar 40 μm). In **(C)** immunoblotting mediated quantification of Itgb1 shows severe downregulation in IM from Itgb1^f/f^-Aqp2^cre/+^ mice. Data are expressed as mean ± sem; n power is 5 vs. 5, ^∗∗∗^*p* < 0.001 (unpaired *t*-test). **(D)** shows data from mRNA quantification by qPCR both in the CTX and IM. Data are expressed as mean ± sem; n power is 5 vs. 5, ^∗∗^*p* < 0.01, ^∗^*p* < 0.05 (unpaired *t*-test). **(E–H)** show representative pictures of hematoxylin and eosin from 2 months old mice. At low magnification **(E)** Itgb1^f/f^-Aqp2^cre/+^ mice present dilatations in all renal zones (scale bar 250 μm), in addition in the ISOM they lose the typical medullary pattern of alternating vascular and tubular bundles indicated in the panel **G** with T and V **(F–G)** (scale bar 20 μm).

Model validation was carried out as above. Beta galactosidase expression was detected only in *Itgb1*^f/f^-AQP2^cre/+^ mice and was limited to CD distribution pattern (**Figures 4A,B**). A severe downregulation of Itgb1 protein (**Figure 4C**) and mRNA (**Figure 4D**) expression in the IM of *Itgb1*^f/f^-AQP2^cre/+^ mice corroborates recombination efficiency. CDs represent a limited amount of the entire renal cortex cellular population. Thus, since Itgb1 is ubiquitously expressed in all epithelial and non-epithelial cells, only a slight reduction of Itgb1 protein abundance was evaluated from cortical samples, likely sustained by a compensatory upregulation of the mRNA level (**Figure 4D**).

*Itgb1*^f/f^-AQP2^cre/+^ mice were born at Mendelian ratio. They showed no difference from their control littermates at birth and only a tendency toward a reduced body weight at 1 month of age (**Supplementary Table S2**). No differences in kidney over body weight ratio, neither major phenotypical alterations in the early post-natal period suggest that no developmental alterations of the CDs occur in *Itgb1*^f/f^-AQP2^cre/+^. However, significant difference in body weight was detected at 2 months together with the development of overt hyposmotic polyuria and polydipsia (**Supplementary Table S2**).

At microscopical level, this polyuric phenotype is associated with dilatation of CDs and upstream segments (**Figures 4F,H**) resembling the same histological pattern secondary to bilateral ureteral obstruction (**Supplementary Figure S1**; Matsell et al., 2002). No tubular dilatations are observed in control mice of same age (**Figures 4E,G**). However, this pattern is not associated with signs of hydronephrosis, suggesting that tubular collapse could occur. With time this polyuric condition leads to renal failure and then reduced lifespan: in fact *Itgb1*^f/f^-AQP2^cre/+^ mice die around 90 days after birth.

Finally, at 1 and 2 months of age, no variations in serum sodium, potassium and chloride reveals that, despite the severe polyuria, *Itgb1*^f/f^- AQP2^cre/+^ mice are still able to maintain renal electrolyte homeostasis (**Supplementary Table S2**).

### Itgb1 Ablation in AQP2 Positive Cells Leads to Nephrogenic Diabetes Insipidus

**FIGURE 5:**
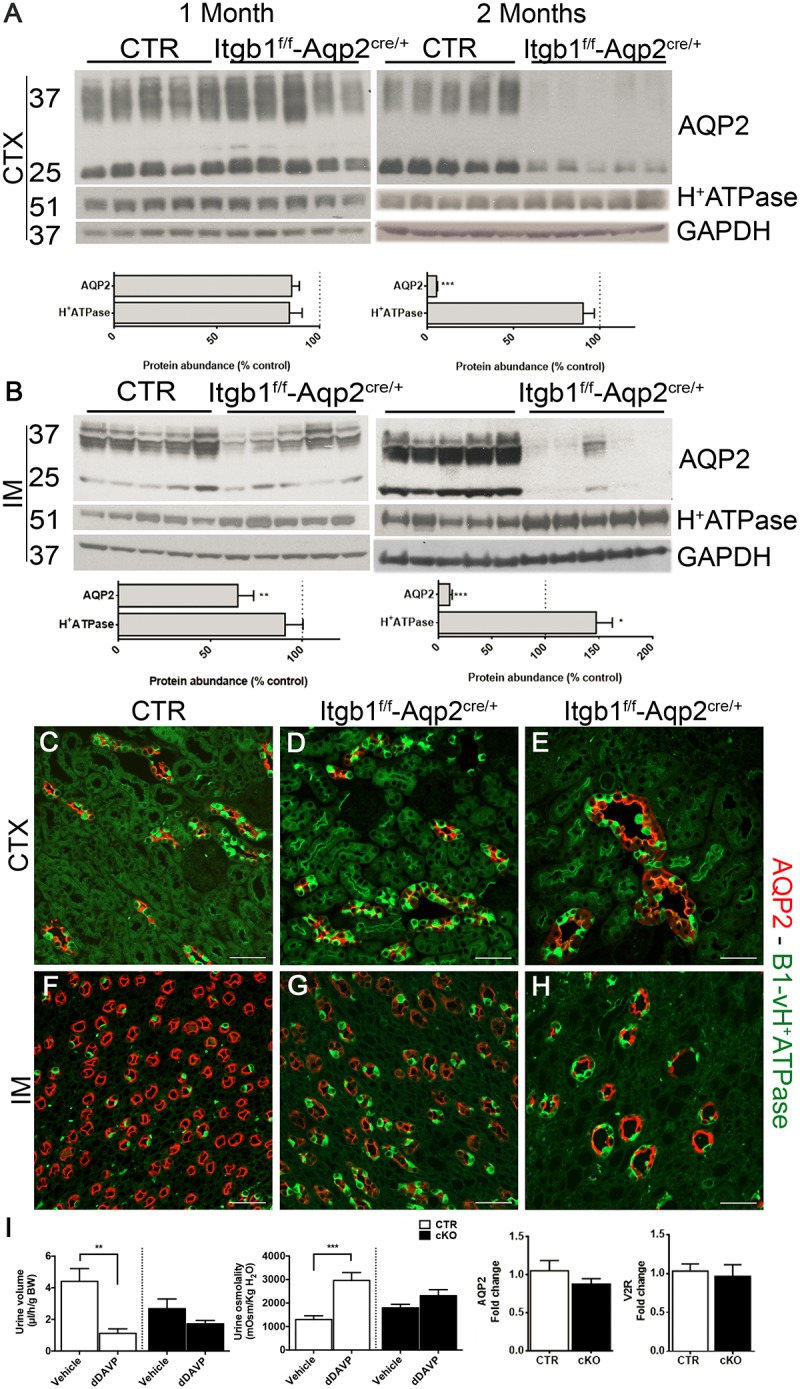
Suppression of Itgb1 b1 affect collecting duct structure. In **(A,B)** immunoblotting shows the relative abundance of cortical and medullary AQP2 and B1-vH^+^ATPase in 1 and 2 months old mice. AQP2 expression is significantly reduced in IM already at 1 month and then also in the CTX in 2 months in Itgb1^f/f^-Aqp2^cre/+^ mice compared to CTR. This is paralleled by an increased relative abundance of B1-vH^+^ATPase in IM. Data are expressed as mean ± sem; n power is 5 vs. 5. ^∗^*p* < 0.05, ^∗∗^*p* < 0.01, ^∗∗∗^*p* < 0.001. In **(C–H)** representative pictures of the CTX and IM from 1 and 2 months mice labeled with an anti-AQP2 (red) and anti-B1-vH^+^ATPase (green) antibodies corroborate the lower density of collecting ducts in Itgb1^f/f^-Aqp2^cre/+^ mice compared to CTR. In **(I)**, 1 month old mice are challenged with dDAVP administration. At this stage, even though the polyuric phenotype is not manifested yet and no variation in the expression of AQP2 and V2R occurred, Itgb1^f/f^-Aqp2^cre/+^ fail to maximally concentrate urine compared to CTR.

To assess the underlying molecular mechanisms behind the progressive development of the hyposmotic polyuria, at first we focused on the CDs, evaluating the expression profile of the two functional marker proteins of intercalated and principal cells, namely subunit B1 of vH^+^-ATPase and AQP2, respectively. At 1 month of age when no significant differences in urine output and osmolality were detectable between *Itgb1*^f/f^-AQP2^cre/+^ and control littermates, there were no variations in abundance of AQP2 and subunit B1 of vH^+^-ATPase in CTX (**Figure 5A**). However, in 1 month old mice, a downregulation of AQP2 occurred in IM (**Figure 5B**). These medullary alterations worsen in 2 months old mice, in which overt polyuria (**Supplementary Table S2**) paralleled a severe downregulation of AQP2 also in CTX (**Figure 5A**) and a likely compensatory upregulation of subunit B1 of vH^+^-ATPase. Double staining with anti-AQP2 and anti B1-vH^+^ATPase (**Figures 5C–H**) or anti-AQP2 and anti-AQP4 antibodies (**Supplementary Figure S3**), corroborates these data showing that downregulation of AQP2 in *Itgb1*^f/f^-AQP2^cre/+^ mice was due to disappearance of medullary CDs with time. Downregulation of ENaC expression (**Supplementary Figure S2**) confirmed that Itgb1 is crucial for the overall principal cells function and not only for AQP2 system.

In order to evaluate early signs of CDs dysfunction, we challenged non-polyuric yet 1 month old mice with dDAVP or vehicle. Acute dDAVP administration promotes water concentration by stimulating the V2R–AQP2 axis in principal cells. Thereafter, we validated that *Itgb1*^f/f^-AQP2^cre/+^ mice presented no significant difference in AQP2 and V2R mRNA expression compared to control littermates (**Figure 5I**). While control mice properly increase their urinary osmolality and decrease urine volume after 5 h from dDAVP administration, no similar changes were detected in *Itgb1*^f/f^-AQP2^cre/+^ mice, revealing a resistance to dDAVP. Finally, the similar urinary osmolality both at baseline (**Supplementary Table S2**) and after vehicle injection reveals that no changes in the interstitial osmolality were present in 1 month old *Itgb1*^f/f^-AQP2^cre/+^ mice and supports the integrity of the counter-current multiplication mechanisms at this stage.

### Selective Suppression of Itgb1 in PC Alters NKCC2 Expression in the TAL

**FIGURE 6:**
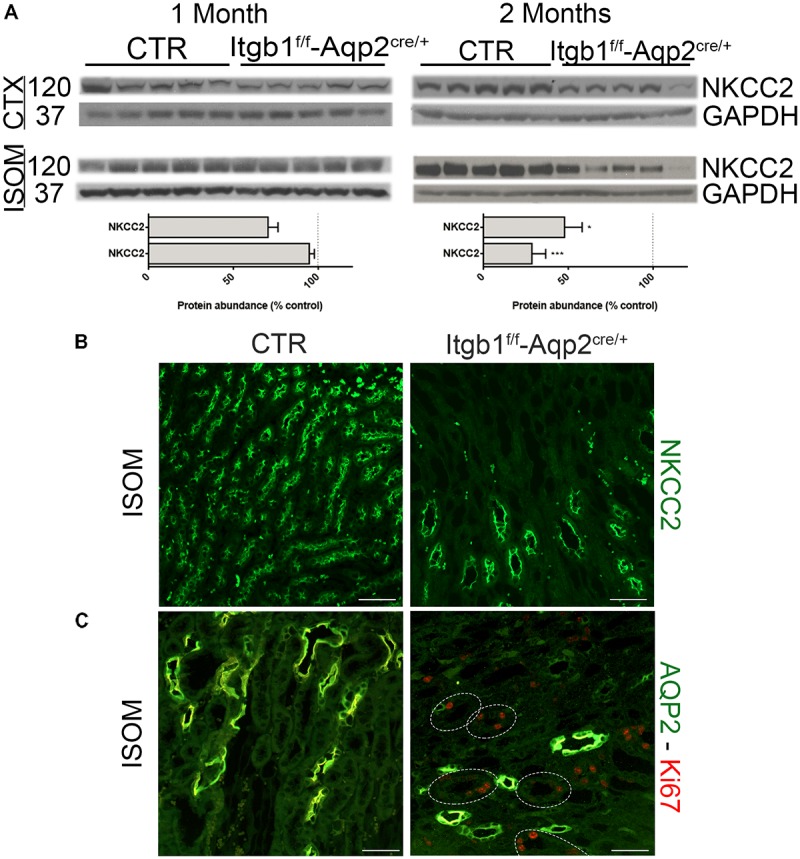
Selective ablation of Itgb1 in the collecting duct affect NKCC2 structure as well. In **(A)** immunoblotting shows the relative abundance of cortical and medullary NKCC2 in 1 and 2 months old mice. Itgb1^f/f^-Aqp2^cre/+^ mice showed a significant downregulation of cortical and medullary NKCC2 at 2 months. Data are expressed as mean ± sem; n power is 5 vs. 5. ^∗^*p* < 0.05 and ^∗∗∗^*p* < 0.001. In **(B)** representative pictures of renal ISOM stained with anti- NKCC2 antibody, show a lower density of TAL segment in 2 months old Itgb1^f/f^-Aqp2^cre/+^ mice. In **(C)** representative pictures of renal ISOM labeled with anti-AQP2 (green) and anti-Ki67 (red) antibodies show an increase proliferation rate in TAL (AQP2 negative segments).

The counter-current multiplication mechanism is necessary to accumulate osmolites in the medullary interstitium and so to generate the osmotic gradient to finally reabsorb water along the CDs. The TAL is the crucial segment for this mechanism (Carota et al., 2010), to the point that hereditary conditions like Bartter syndrome, characterized by defective TAL function, are addressed as inherited secondary nephrogenic diabetes insipidus (Bockenhauer et al., 2012). In order to evaluate whether Itgb1 deletion causes NDI exclusively by targeting the CD, we examined NKCC2 expression level and localization. As showed in **Figure 6A**, at 1 month of age there was no variation in neither cortical or medullary NKCC2 abundance in *Itgb1*^f/f^-AQP2^cre/+^ mice compared with control.

However, at 2 months of age a significant downregulation of NKCC2 was detected both in the CTX and in the ISOM of *Itgb1*^f/f^-AQP2^cre/+^ mice. Staining with an anti-NKCC2 antibody of renal ISOM still prove apical localization of the protein and confirms lower density of the TAL in 2 months old *Itgb1*^f/f^-AQP2^cre/+^ mice. These results match with the progressive loss of CDs and suggest a connection between these events. Indeed, in 2 months old *Itgb1*^f/f^-AQP2^cre/+^ mice, the typical morphological pattern of ISOM with vascular bundles alternating to CD and TAL bundles (Zhai et al., 2006) is completely lost (**Figure 6B**). Suppression of Itgb1 along the CD is able to influence TAL morphology and proliferation. Indeed, we found an increased proliferation rate (Ki67 positive cells) in medullary TAL of *Itgb1*^f/f^-AQP2^cre/+^ mice (**Figure 6C**). These findings corroborate the fact that, even though selective for CD, Itgb1 suppression influences also TAL morphology and function at the same extent as in *Itgb1*^f/f^-*Pax8*^cre/+^ mice (**Supplementary Figure S4**).

## Materials and Methods

### Transgenic Mouse Generation and Animal Experiments

*Itgb1*^f/f^-*Pax8*^cre/+^ mice were generated by breeding together β1-Integrin^f/f^ mice (Brakebusch et al., 2000) with *Pax8*^cre/+^ mice (Bouchard et al., 2004); *Itgb1*^f/f^-*Pax8*^+/+^ mice served as control.

*Itgb1*^f/f^-AQP2^cre/+^ mice were generated by using AQP2^cre/+^ mice (Ronzaud et al., 2007) instead of the *Pax8*^cre/+^ line; *Itgb1*^f/f^-AQP2^+/+^ mice served as control. A scheme showing the recombination strategy and the survival rate of the single transgenic model is reported in **Supplementary Figure S5**.

Genotyping was performed by PCR analysis of ear biopsy. The following primers were used:

*Itgb1:* L1: 5′-GTGAAGTAGGTGAAAGGTAAC-3′; T56: 5′-AGGTGCCCTTCCCTCTAGA-3′.
Pax8: E3: 5′-CCCTCCTAGTTGATTCAGCCC-3′; I2: 5′TCTC CACTCCAACATGTCTGC-3′; CG: 5′-AGCTGGCCCAAAT GTGCTGG-3′ (*I2 and E3 were used for the wt allele, I2 and CG for the Cre allele*).
Aqp2: P1: 5′-AAGTGCCCACAGTCTAGCCTCT-3′; P2: 5′-CCTGTTGTTCAGCTTGCACCAG-3′; P3: 5′-GGAGAACG CTATGGACCGGAGT-3′ (*P1 and P2 were used for the wt allele, P1, and P3 for the Cre allele*).

Metabolic parameters were collected as previously reported (Reale et al., 2016). dDAVP administration test was performed according to Trepiccione et al. (2016a). Soon after voiding the bladder on a cold plate, mice were injected i.p. with vehicle (0.9% NaCl, 5 ml/Kg) or 1 μg/kg of body weight dDAVP. Then urine was collected after 5 h in metabolic cages.

FITC-Inulin clearance was performed as previously described (Damiano et al., 2013; Iervolino et al., 2015). Briefly, mice were anesthetized with Inactin (Sigma, Milan, Italy) 100 mg/kg of BW, tracheostomized, and placed on the temperature-controlled surgical table. The left jugular vein was cannulated with a PE-10 catheter for infusion via a syringe pump (Infusion Pump High Tech, KDS Legato 200, Sigma Milan, Italy). The right carotid artery was catheterized to monitor blood pressure *via* a blood pressure recorder (BP1 by WPI, Sarasota, FL, United States) and for blood samples collection. The bladder was catheterized with a PE-50 tube for urine collection. Subsequently, mice received a constant infusion of FITC-inulin (Sigma, Milan, Italy) at a rate of 0.15 μl/min/g of BW. After 60 min of equilibration, urine samples were collected every 30 min for the following 2 h. Glomerular filtration rate (GFR) was calculated using standard clearance formula GFR = [U] ^∗^ V/[P]. Serum and urinary FITC-inulin samples were first stabilized in 10 mM HEPES and measured at spectrophotometer (EnVision, 2104-0010A, PerkinElmer, Waltham, MA, United States).

Blood pressure recording was evaluated in mice anesthetized with Inactin. The carotid was catheterized and connected to the blood pressure recorder (BP1 by WPI). All the procedures involving animals were performed according to the Italian Ministry of Health decree nr 100/2006 and later decree 26/2014. *In vivo* experiments were also approved by the local Animal Ethics Committee (CESA) of Biogem (Ariano Irpino, Italy) with ID 4613 and 7917.

### Urine and Blood Analysis

Urinary proteins level was assessed by Bradford assay (Biorad Protein Assay, Biorad, Segrate, Italy). Urine osmolality was measured by freezing point depression osmometer (Model 3320, Advanced Instruments, inc., MA, United States) as previously reported (Zacchia et al., 2016). Serum electrolytes were analyzed by Vitrovet (Scil, Treviglio (BG), Italy).

### Histology and Immunofluorescence

Mice were anesthetized with isoflurane, the left kidney was collected for protein and mRNA evaluation. Then, mice were perfused through the abdominal aorta with 4% paraformaldehyde (PFA). After embedding in paraffin, 4 μm thick sections were stained with haematoxylin and eosin (Sigma-Aldrich, Milan, Italy).

Immunofluorescence was performed as previously (Trepiccione et al., 2017). Target retrieval was performed in TEG buffer pH 9.2. Primary antibodies were incubated overnight at 4°C. Secondary antibodies were incubated for 1 h at room temperature. Stained sections were mounted with fluorescent mounting medium (Dako, CA, United States). Axio Observer Z1 (Zeiss, Oberkochen, Germany) was used for the acquisition of images.

β-Galactosidase assay was performed as described in Traykova-Brauch et al. (2008). Perfused and fixed in 4% PFA, kidneys were transferred into 30% sucrose in phosphate buffer (PBS) for 24 h. After embedding in OCT, they were snap-frozen. 5-bromo-4-chloro-3-indolyl-galactopyranoside (X-gal) 1 mg/ml (Sigma-Aldrich, Milan, Italy) was dissolved in DMSO and added to the reaction buffer containing: 0.01% sodium deoxycholate, 2 mM MgCl_2_, NP-40 0.02%, 8 mM potassium ferricyanide, and 8 mM potassium ferrocyanide in PBS (pH 7.4). The frozen sections were incubated at 37°C in the X-gal solution in a humidity controlled chamber over night, dehydrated, and mounted with Eukitt medium (Bio-optica, Milan, Italy).

### Immunoblotting

The kidneys were dissected in Cortex/OSOM, ISOM, and IM. Tissues were homogenized with a Tissue Lyser RETSCH MM300 (Qiagen, Milan, Italy) in the lysis buffer (sucrose 0.3 M, imidazole 25 mM, ethylenediaminetetraacetic acid (EDTA) 1 mM, phenylmethylsulfonyl fluoride (PMSF) 1 mM) with protease (Complate Protease Inhibitor Cocktail, Santa Cruz, Dallas, TX, United States) and phosphatase inhibitor cocktail (PhosSTOP, Roche, Monza, Italy). Total protein concentration was measured by Bradford assay (BioradProtein Assay, Segrate, Italy). SDS-PAGE was performed on NuPage 4–12% Bis-Tris Gel (Waltham, MA, United States) or on homemade gels (stacking gel: 0.5M Tris-HCl pH 6.8, acrylamide/bis 30%, 10% sodium dodecyl sulfate (SDS) 0.1%, ammonium peroxodisulfate (APS) 0.1%, tetramethylethylenediamine (TEMED) 0.1%, resolving gel: 1.5M Tris-HCl pH8.8, 10% SDS0.1%, APS 0.1%, TEMED 0.1%. Proteins were then transferred to polyvinylidene difluoride (PVDF) membranes (Invitrolon PVDF, Invitrogen, Waltham, MA, United States). PVDF membranes were incubated overnight with primary antibodies at 4°C, washed, and subsequently incubated with secondary antibody for 1 h at room temperature. Proteins were visualized using enhanced chemiluminescence (Pierce, ECL Western Blotting, c.a.32106, Thermo Fisher Scientific, Waltham, MA, United States). Densitometry was performed using Image-J software.

### Antibodies

The following primary antibodies were used: anti-AQP2 (7661AP) was kindly provided by Prof. Sebastian Frische; immunofluorescence (IF) and immunoblotting (IB) 1:1,000; rabbit anti-Ki67 (#4203–1, Epitomics, Cambridge, United Kingdom) IF 1:500; anti-B1/2 H^+^ATPase (#sc-55544, Santa Cruz, Dallas, TX, United States) IF and IB 1:1,000; rabbit anti-NKCC2 (#AB3562P, Millipore, Dramstad, Germany) IF and IB 1:1,000; rabbit anti-β1 integrin (#04-1109, Millipore, Dramstad Germany) IB 1:1,000; rabbit anti-ENaC alpha (SPC-403, StressMarq, Victoria, BC, Canada) IB 1:1000; mouse anti-β-Actin (#A2066 Sigma, Milan, Italy) IB 1:20,000; rabbit anti-GAPDH (#10018 GeneTex, Hsinchu City, Taiwan) IB 1:20,000. The following secondary antibodies were used: goat anti mouse Alexa Fluor 488 IF 1/800 (Invitrogen, CA, United States) goat anti-rabbit Cy3 (A10520, Invitrogen, CA, United States) IF 1:500; anti-mouse HRP conjugated (NA931V GE Healthcare, Little Chalfont, United Kingdom) IB 1:2,000; anti-rabbit HRP conjugated (NA934V GE Healthcare, Little Chalfont, United Kingdom) IB 1:2,000.

### RNA Extraction and q-PCR

Total RNA was isolated from tissues using TRIsure reagent (BIOLINE, A Meridian life Science^®^ Company, WilfongRdMemphis, TN, United States) and 1 μg of RNA was reverse-transcribed by Quantitec reverse transcription kit (Qiagen, Milan, Italy) according to the manufacture’s instruction. The Q-PCR was performed with Power PCR Master Mix 16 (Applied Biosystems, Waltham, MA, United States) according to the manufacturer’s instruction and the following primers were used: Itgb1, CCAGCCAAGTGACATAGAGAA (forward) and GGTAATCTTCAGCCCTCTTGA (reverse); *Aqp2*, GCAGTT GTCACTGGCAAGT (forward) and AGGGGAACAGCAGGTA GTTG (reverse); *V2r*, GACTAAGTTGGCCTCCTGTGA (forward) and GGTCTCGGTCATCCAGTAGC (reverse); *Gapdh*, CTGTGGATGGCCCCTCTGGA (forward) and GGGCCCTCAGATGCCTGCTT (reverse). Reactions were run on a 7900HT system (Applied Biosystems, Waltham, MA, United States). GAPDH was used for normalization.

### Statistics

All data are presented as mean ± SEM. Statistical analysis was performed by an unpaired *t*-test. A value of *p* < 0.05 was considered statistically significant.

## Discussion

Our findings show that Itgb1 is necessary for the function of renal epithelial cells and for the overall architecture of the ISOM. By generating 2 mouse model selective cKO for Itgb1 we found that the medullary tubular bundles composed by the TAL and the CD are connected together by Itgb1 expression and it is sufficient to suppress Itgb1 just in the CD cells to alter TAL morphology and function as well.

Integrins mediate the cell–extracellular matrix (ECM) interactions. On the basolateral side, they anchor cells to the ECM. However, this is not a static interaction, but it mediates a complex of “inside-out” and “outside-in” signals that link cell function to the extracellular space. This makes integrins an important group of protein in organogenesis, but also in cellular migration and tissue repairing (Graber et al., 1999). Itgb1 constitutes the majority of heterodimers combinations of the integrin complex and it is, by far, among the most abundant in the kidneys. It is critical for both kidney and CD tree development (Wu et al., 2009; Zhang et al., 2009). Indeed, the development of the CD is a finely tuned process of growth and branching of tubules requiring a continuous interaction between cells and the surrounding ECM.

The intimate relation between cells and ECM is critical for renal function, not only for glomeruli, but, above all, for tubules. Indeed, tubule shape and conformation respond to specific functional needs. This is the case for typical U-shaped architecture of loop of Henle and vasa recta, that allows the generation of zones with different interstitial osmolality and oxygen tension. This is crucial for the development of the counter current mechanism and water reabsorption, on one side preventing interstitial osmole wash-out, and, on the other side, exposing the medulla to hypoxic stress. The equilibrium point is kept by a the fine and highly conserved architecture of the medulla resulting from an alternation of vascular and tubular bundles (Zhai et al., 2006; Wei et al., 2015). Tubular bundles in the ISOM are made by TAL and CD strictly connected to each other by a slight layer of ECM. In this scenario, it is not peregrine to think that any alteration of these connections can potentially be harmful.

We showed here that the suppression of Itgb1 in renal epithelial cells leads to a general impairment of renal function including an increasingly worsening proteinuria and urinary concentrating defect, ending up to severely low GFR and hypertension. Since post-natal mice do not present severe renal abnormalities and they pass the weaning, no defects in nephrogenesis should account for it.

*Itgb1*^f/f^-*Pax8*^cre/+^ mice develop overt proteinuria and severe renal failure at the same extent as showed in podocytes specific Itgb1 cKO mice (Pozzi et al., 2008). The connection between podocytes and basement membrane is crucial for filtration barrier integrity. Loss of expression of integrins alters the function of podocytes, contributing to their detachment from the basement membrane (Pozzi et al., 2008; Girgert et al., 2010). Our *Itgb1*^f/f^-*Pax8*^cre/+^ mice model presents overt proteinuria and a similar histological pattern as the podocyte specific cKO model (Pozzi et al., 2008), suggesting a similar pathogenic mechanism.

In addition, *Itgb1*^f/f^-*Pax8*^cre/+^ mice developed severe polyuria with moderately low urine osmolality. This was associated with distal nephron dysfunction and, in particular, with downregulation of AQP2 and NKCC2 expression. However, since the expression of PAX8 promoter is effective also in the PT, we could not exclude an impaired water reabsorption at this level as showed in PT specific Itgb1 suppression (Elias et al., 2014). *Itgb1*^f/f^-*Pax8*^cre/+^ mice develop progressive renal failure. Since this condition is related to urinary concentration defect by downregulation of AQP2 and NKCC2 (Amlal et al., 2003) it was not possible to assess whether polyuria was a primary Itgb1 related effect.

In order to address this point, we generated a AQP2 specific Itgb1 cKO mouse model. These mice present severe polyuria associated to severe AQP2 downregulation and progressive loss of CD. Our results confirm previous findings from Mamuya et al. (2017). In a similar model, Itgb1 suppression in AQP2 positive cells leads to the apoptosis of PC as showed by caspase 3 staining (Mamuya et al., 2017). In our model, we provided evidences of a progressive impairment of urinary concentration ability that becomes evident in 2 months old mice, but it is anticipated at 1 month by resistance to dDAVP administration. Dysfunction of the CD begins with AQP2 downregulation in the cortex and it then extents to inner medulla at 2 months. At this stage, *Itgb1*^f/f^-*AQP2*^Cre/+^ mice present a severe tubular dilatation that extends to the entire proximal nephron and glomerular expansion, resembling a model of bilateral ureteral obstruction (Matsell et al., 2002). Since no signs of hydronephrosis are present, this phenotype is likely due to tubular collapse and CD disappearance. Indeed, suppression of Itgb1 severely affects the PC leading to similar finding as in *anoikis* (Taddei et al., 2012). However, surprisingly, in parallel with CD dysfunction, we also showed a progressive downregulation of NKCC2, the functional marker of TAL and a disappearance of this type of segment. This seems not related to off-target CRE-LOX recombination of the *Itgb1*^f/f^-*AQP2*^cre/+^ mice since, as showed by beta-GAL staining, no recombination occurred in the TAL. In addition, we recently showed that AQP2 positive cells can give rise to some intercalated and DCT cells, but not to TAL cells (Trepiccione et al., 2016b). Residual TAL segments in the ISOM presented with dilatation and increased cells number. When screened for ki67 proliferation markers TAL cells of *Itgb1*^f/f^- *AQP2*^cre/+^ mice were highly positive compared to controls. Altogether these findings lead to the alteration of the normal histological pattern of the ISOM with disappearance of the typical division in vascular and tubular bundles (Zhai et al., 2006). These results suggest that CD and TAL are intimately connected to each other through ECM contact. A similar thigh interaction between renal cell and ECM has been demonstrated for B-type intercalated cells. Suppression of Itgb1 in these cell type alters the interaction with the extracellular component of the ECM, hensin, and it impairs the transition from B- to A-type intercalated cells after acid challenge (Gao et al., 2010).

Itgb1 is a component of many heterodimers complexes which present a different specificity with ECM components. A potential explanation of the observed time-related phenotype development, progressively from 1 month of age onwards, could be ascribed in part to age-related changes in ECM composition. Indeed, since PAX8 and AQP2 expression occurs in pre-natal stage and proven recombination efficacy was already detected in 1 month old transgenic mice (beta-GAL staining), it seems unlikely that late onset recombination determines the phenotype. A possible age-related change in the composition of the medullary ECM could have triggered the development of the phenotype. Several evidences showed that the composition of ECM components change both in the glomeruli and in medullary interstitium (Abrass et al., 1995). In this speculative scenario, the susceptibility of renal cells to loss of expression of Itgb1 could depend also by the specific composition of the ECM.

Here we showed that Itgb1 is crucial for all the renal epithelial cells and that the suppression of Itgb1 exclusively in the PC is sufficient for secondarily altering TAL structure and function. On one side, the Itgb1 suppression leads to PC *anoikis*, while on the other side it induces TAL cells proliferation and dysfunction. Here we provide evidences that cell to cell interaction is not a prerogative of the apical membrane process sustained by messengers released in the urine, but also of the basolateral membrane promoted through the Itgb1. This latter is critical for the architecture and function of the ISOM and could be ascribed as an additional mechanism of cell to cell crosstalk.

## Author Contributions

FT, AI, GC, MDF, and AP conception and design of research. AI, FT, LDLM, FPe, GS, and FPr performed the experiments. AI, FT, DDM, GC, MDF, and AP analyzed the data. FA contributed to the generation of the mouse model *PAX8-Itgb1* cKO.

## Conflict of Interest Statement

The authors declare that the research was conducted in the absence of any commercial or financial relationships that could be construed as a potential conflict of interest. The reviewer MA-b and handling Editor declared their shared affiliation.
