# Corrigendum: Integrin Beta 1 Is Crucial for Urinary Concentrating Ability and Renal Medulla Architecture in Adult Mice

**DOI:** 10.3389/fphys.2018.01676

**Published:** 2018-11-29

**Authors:** Anna Iervolino, Luigi R. De La Motte, Federica Petrillo, Federica Prosperi, Francesca Maria Alvino, Guglielmo Schiano, Alessandra F. Perna, Danilo Di Matteo, Mario De Felice, Giovambattista Capasso, Francesco Trepiccione

**Affiliations:** ^1^Biogem Scarl, Istituto di Ricerche Gaetano Salvatore, Ariano Irpino, Italy; ^2^Department of Translational Medical Sciences, University of Campania “Luigi Vanvitelli”, Naples, Italy; ^3^Department of Molecular Medicine and Medical Biotechnologies, University of Naples “Federico II”, Naples, Italy

**Keywords:** integrin beta1, collecting duct, thick ascending limb, renal failure, AQP2

In the original article, Francesca Maria Alvino was not included as an author in the published article. The authors apologize for this error and state that this does not change the scientific conclusions of the article in any way. The original article has been updated.

## Conflict of interest statement

The authors declare that the research was conducted in the absence of any commercial or financial relationships that could be construed as a potential conflict of interest.

