# Supplementary material for: Integrin Beta 1 Is Crucial for Urinary Concentrating Ability and Renal Medulla Architecture in Adult Mice
Source: Front Physiol. 2018 Sep 13;9:1273. doi: 10.3389/fphys.2018.01273 (PMC6147158; doi:10.3389/fphys.2018.01273)
Supplement: Supplementary file 1 [file Table_1.docx]

**Supplementary Material**

Supplementary tab-1

|  |  | m of age | **Itgb1^f/f^** | | | | **Itgb1^f/f^ -Pax8^cre/+^** | | | |  |
| --- | --- | --- | --- | --- | --- | --- | --- | --- | --- | --- | --- |
| Body weight | g | 1 | 18.9 | ± | 1.4 | (4) | 17.46 | ± | 0.6 | (4) |  |
|  |  | 3 | 26.5 | ± | 0.6 | (4) | 22.8 | ± | 0.5 | (5) | ** |
| Kw/ Bw | g/g | 1 | 1.22 | ± | 0.05 | (4) | 1.05 | ± | 0.12 | (4) |  |
|  |  | 3 | 1.28 | ± | 0.05 | (4) | 0.93 | ± | 0.06 | (5) | ** |
| Proteinuria | mg/24h | 1 | 0.73 | ± | 0.23 | (4) | 1.71 | ± | 0.12 | (7) | ** |
|  |  | 3 | 2.87 | ± | 0.26 | (4) | 5.36 | ± | 0.68 | (9) | * |
| Systolic Blood Pressure | mmHg | 1 | 94 | ± | 3.27 | (5) | 94 | ± | 0.75 | (5) |  |
|  |  | 3 | 93 | ± | 2.04 | (5) | 127 | ± | 3.33 | (5) | *** |

Physiological parameters of Itgb1-Pax8 cKO mice and their relative controls. In the brackets n-power, data are expressed as mean ± standard errors * is for p-value < 0.05; ** is for p-value < 0.01; *** is for p-value < 0.001.

Supplementary Tab-2

|  |  | m of age | **Itgb1^f/f^** | | | | **Itgb1^f/f^ -Aqp2^cre/+^** | | | |  |
| --- | --- | --- | --- | --- | --- | --- | --- | --- | --- | --- | --- |
| Body weight | g | 1 | 16.3 | ± | 0.56 | (11) | 14.6 | ± | 0.62 | (20) |  |
|  |  | 2 | 23.1 | ± | 1.22 | (4) | 16.9 | ± | 0.38 | (11) | *** |
| Kw/ Bw | g/g | 1 | 1.13 | ± | 0.06 | (7) | 1.15 | ± | 0.04 | (16) |  |
|  |  | 2 | 1.13 | ± | 0.06 | (4) | 1.25 | ± | 0.05 | (6) |  |
| [Na+] | mM | 1 | 149 | ± | 0.75 | (4) | 147 | ± | 0.73 | (5) |  |
|  |  | 2 | 147 | ± | 1.46 | (4) | 153 | ± | 2.65 | (6) |  |
| [K+] | mM | 1 | 5.05 | ± | 0.35 | (4) | 5.04 | ± | 0.34 | (5) |  |
|  |  | 2 | 4.25 | ± | 0.23 | (4) | 4.40 | ± | 0.30 | (6) |  |
| [Cl-] | mM | 1 | 119 | ± | 0.40 | (4) | 118 | ± | 0.84 | (5) |  |
|  |  | 2 | 115 | ± | 1.78 | (4) | 115 | ± | 1.75 | (6) |  |
| BUN | mg/dl | 1 | 10.2 | ± | 1.28 | (3) | 10.4 | ± | 1.06 | (6) |  |
|  |  | 2 | 13.9 | ± | 2.85 | (4) | 41.2 | ± | 8.32 | (5) | * |
| Creatinine | mg/dl | 1 | 0.57 | ± | 0.18 | (3) | 0.49 | ± | 0.04 | (6) |  |
|  |  | 2 | 0.71 | ± | 0.08 | (4) | 0.98 | ± | 0.18 | (6) |  |
| Urine Output | µl/h/gbw | 1 | 1.64 | ± | 0.25 | (15) | 1.78 | ± | 0.35 | (25) |  |
|  |  | 2 | 2.41 | ± | 0.35 | (13) | 16.48 | ± | 1.22 | (17) | *** |
| Urine Osmolality | mOsm/Kg H_2_O | 1 | 2198 | ± | 105.5 | (6) | 2463 | ± | 325 | (6) |  |
|  |  | 2 | 2297 | ± | 232 | (6) | 408 | ± | 42 | (6) | * |

Physiological and serum electrolytes, BUN and creatinine of Itgb1-AQP2 cKO mice and their relative controls. In the brackets n-power, data are expressed as mean ± standard errors. * is for p-value < 0.05; *** is for p-value < 0.001

**Supplementary Figure Legend**

**Fig-1S: Itgb1^f/f^-Aqp2^cre/+^mice present tubular dilatation both in inner medulla and cortex**

Representative pictures of hematoxylin and eosin from 2 months old mice showed severe tubular dilatation both in the IM and cortex in Itgb1^f/f^-Aqp2^cre/+^ mice. These structural alterations resemble the typical lesion occurring in bilateral ureteral obstruction. (Scale bar 250μm).

**Fig-2S: Itgb1^f/f^-Aqp2^cre/+^mice present a dowregulation of cortical ENaC expression**

Immunoblotting shows the relative abundance of cortical ENaC expression. Itgb1^f/f^-Aqp2^cre/+^ mice showed a significant downregulation of both 90 KD and 30 KD ENaC, suggesting a general dysfunction of principal cells secondary to Itgb1 suppression. Data are expressed as mean ± sem; n power is 6 vs 6. * is for p value <0.05 ** is for p value <0.01, unpaired t-test.

**Fig-3S: double labelling AQP2-AQP4 confirm a loss of CD in Itgb1^f/f^-Aqp2^cre/+^ cKO mice**

Representative pictures of IM from 1 and 2 months mice labelled with an anti-AQP2 (green) and anti-AQP4 (red) antibodies corroborate the progressive lower density of collecting ducts in Itgb1^f/f^-Aqp2^cre/+^ mice compared to CTR.

**Fig-4S:** **double labelling AQP2-Ki67 single channels**

Representative pictures of renal ISOM labeled with anti-AQP2 (green) and anti-Ki67 (red) antibodies. This is a single channel overview of Fig-6C. AQP2 signal intensity is so strong to be detected also in the red channel acquisition window. High concentration of anti-AQP2 antibody was necessary to detect signal in the remnant CD of Itgb1^f/f^-Aqp2^cre/+^ cKO mice. White circles identified tubules with thick epithelium and negative for AQP2 staining strongly suggesting the TAL since the pictures are acquired from ISOM (inner stripe of outer medulla).

**Fig-5S:** **Schematic representation of used recombination strategy**

On the left, Itgb1f/f-Pax8cre/+ mice were generated by breeding together Itgb1f/f mice (Brakebusch et al., 2000) with Pax8cre/+ mice (Bouchard et al., 2004); on the bottom, the survival curve of this model shows that 80% of the mice dies around 30 weeks from the birth.

On the right, Itgb1f/f-AQP2cre/+ mice were generated by using AQP2cre/+ mice (Ronzaud et al., 2007) instead of the Pax8cre/+ mice); on the bottom, the survival curve of this model shows that 80% of the mice dies around 15 weeks from the birth.
