# Supplementary material for: Integrin Beta 1 Is Crucial for Urinary Concentrating Ability and Renal Medulla Architecture in Adult Mice
Source: Front Physiol. 2018 Sep 13;9:1273. doi: 10.3389/fphys.2018.01273 (PMC6147158; doi:10.3389/fphys.2018.01273)
Supplement: Supplementary file 2 [file Table_2.DOCX]

Supplementary Tab-2

|  |  | m of age | **Itgb1^f/f^** | | | | **Itgb1^f/f^ -Aqp2^cre/+^** | | | |  |
| --- | --- | --- | --- | --- | --- | --- | --- | --- | --- | --- | --- |
| Body weight | g | 1 | 16.3 | ± | 0.56 | (11) | 14.6 | ± | 0.62 | (20) |  |
|  |  | 2 | 23.1 | ± | 1.22 | (4) | 16.9 | ± | 0.38 | (11) | *** |
| Kw/ Bw | g/g | 1 | 1.13 | ± | 0.06 | (7) | 1.15 | ± | 0.04 | (16) |  |
|  |  | 2 | 1.13 | ± | 0.06 | (4) | 1.25 | ± | 0.05 | (6) |  |
| [Na+] | mM | 1 | 149 | ± | 0.75 | (4) | 147 | ± | 0.73 | (5) |  |
|  |  | 2 | 147 | ± | 1.46 | (4) | 153 | ± | 2.65 | (6) |  |
| [K+] | mM | 1 | 5.05 | ± | 0.35 | (4) | 5.04 | ± | 0.34 | (5) |  |
|  |  | 2 | 4.25 | ± | 0.23 | (4) | 4.40 | ± | 0.30 | (6) |  |
| [Cl-] | mM | 1 | 119 | ± | 0.40 | (4) | 118 | ± | 0.84 | (5) |  |
|  |  | 2 | 115 | ± | 1.78 | (4) | 115 | ± | 1.75 | (6) |  |
| BUN | mg/dl | 1 | 10.2 | ± | 1.28 | (3) | 10.4 | ± | 1.06 | (6) |  |
|  |  | 2 | 13.9 | ± | 2.85 | (4) | 41.2 | ± | 8.32 | (5) | * |
| Creatinine | mg/dl | 1 | 0.57 | ± | 0.18 | (3) | 0.49 | ± | 0.04 | (6) |  |
|  |  | 2 | 0.71 | ± | 0.08 | (4) | 0.98 | ± | 0.18 | (6) |  |
| Urine Output | µl/h/gbw | 1 | 1.64 | ± | 0.25 | (15) | 1.78 | ± | 0.35 | (25) |  |
|  |  | 2 | 2.41 | ± | 0.35 | (13) | 16.48 | ± | 1.22 | (17) | *** |
| Urine Osmolality | mOsm/Kg H_2_O | 1 | 2198 | ± | 105.5 | (6) | 2463 | ± | 325 | (6) |  |
|  |  | 2 | 2297 | ± | 232 | (6) | 408 | ± | 42 | (6) | * |
