# Supplementary figures and images for: Integrin Beta 1 Is Crucial for Urinary Concentrating Ability and Renal Medulla Architecture in Adult Mice
Source: Front Physiol. 2018 Sep 13;9:1273. doi: 10.3389/fphys.2018.01273 (PMC6147158; doi:10.3389/fphys.2018.01273)

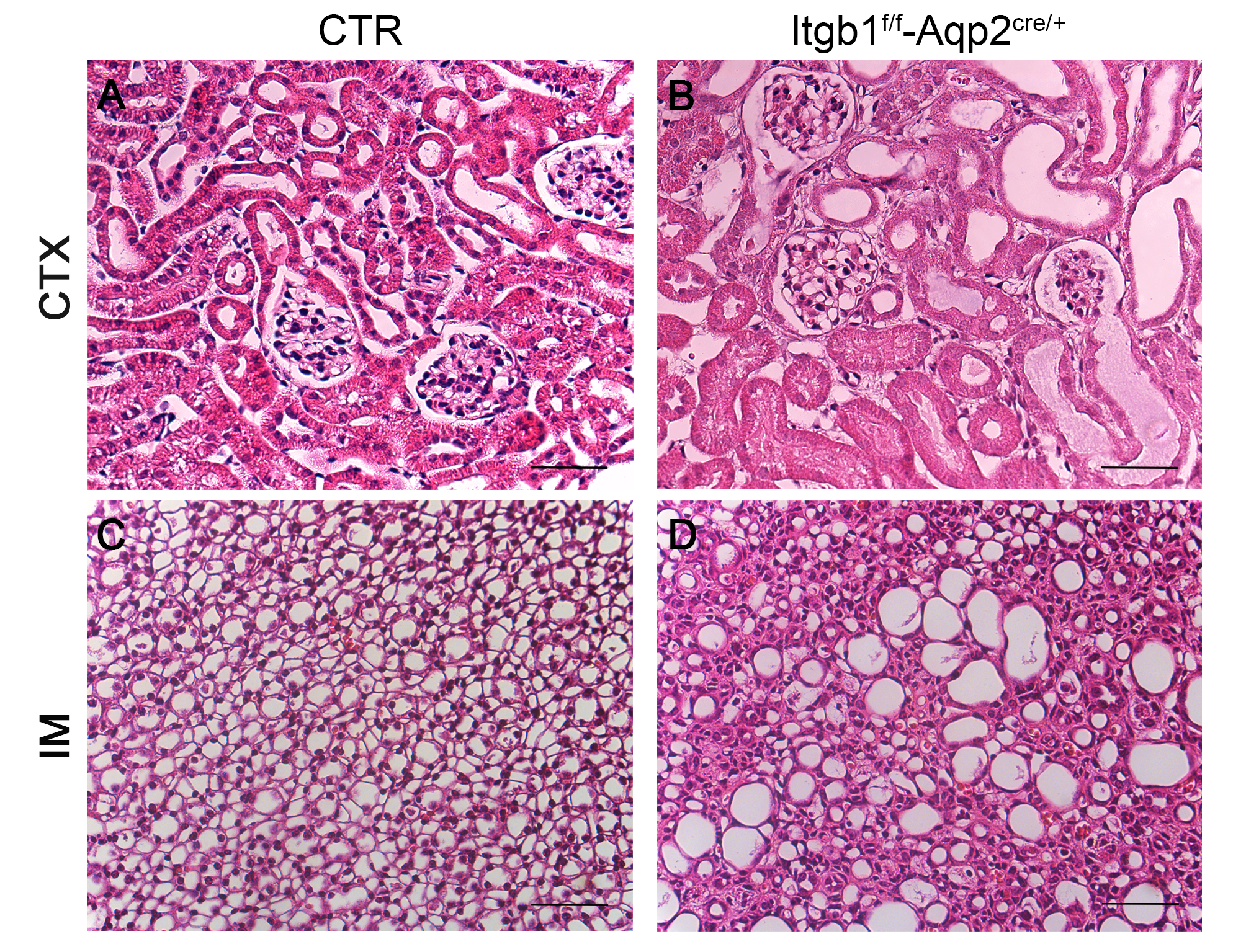

Supplement: Supplementary file 3 [file Image_1.TIF]

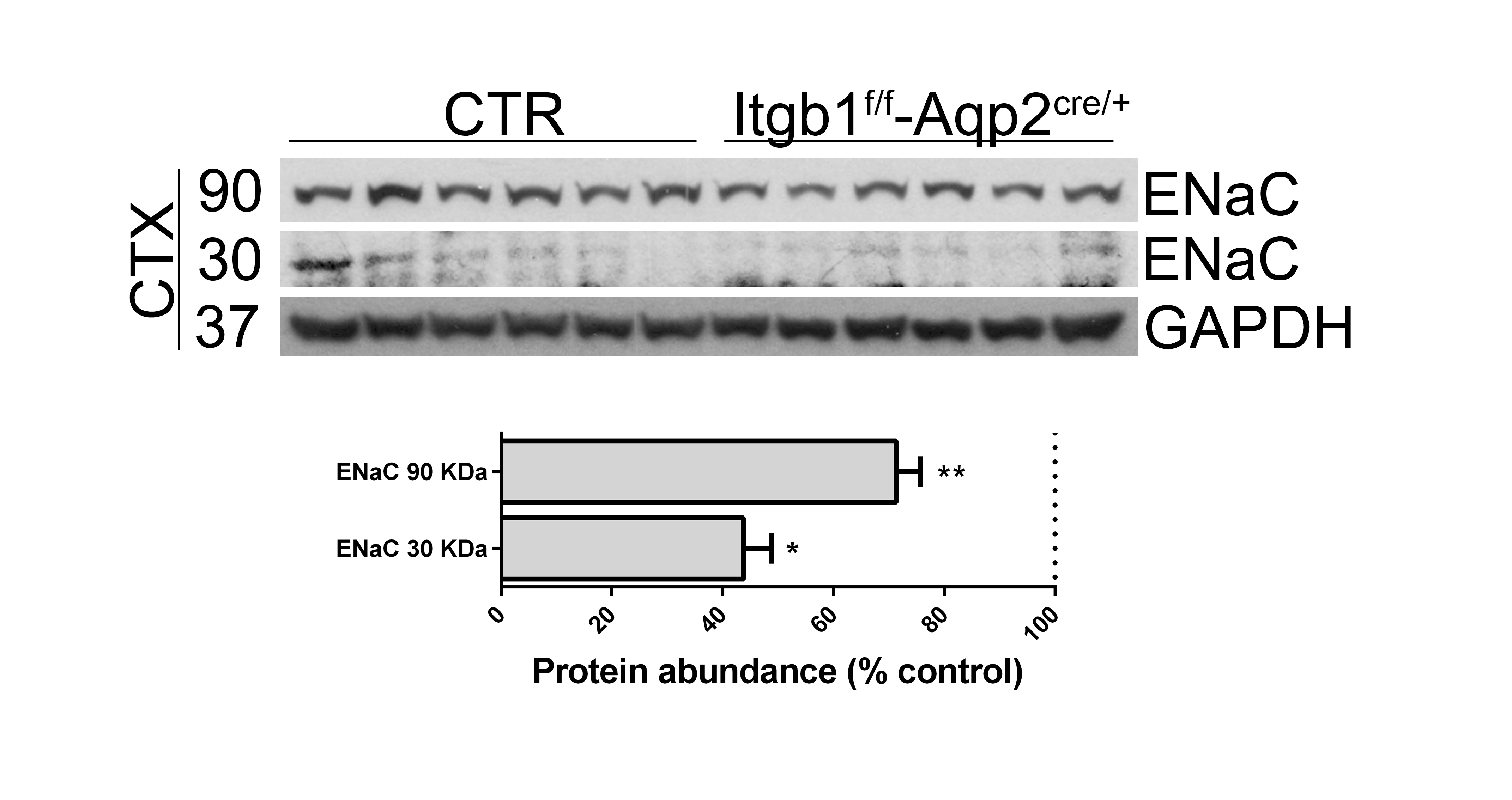

Supplement: Supplementary file 4 [file Image_2.TIF]

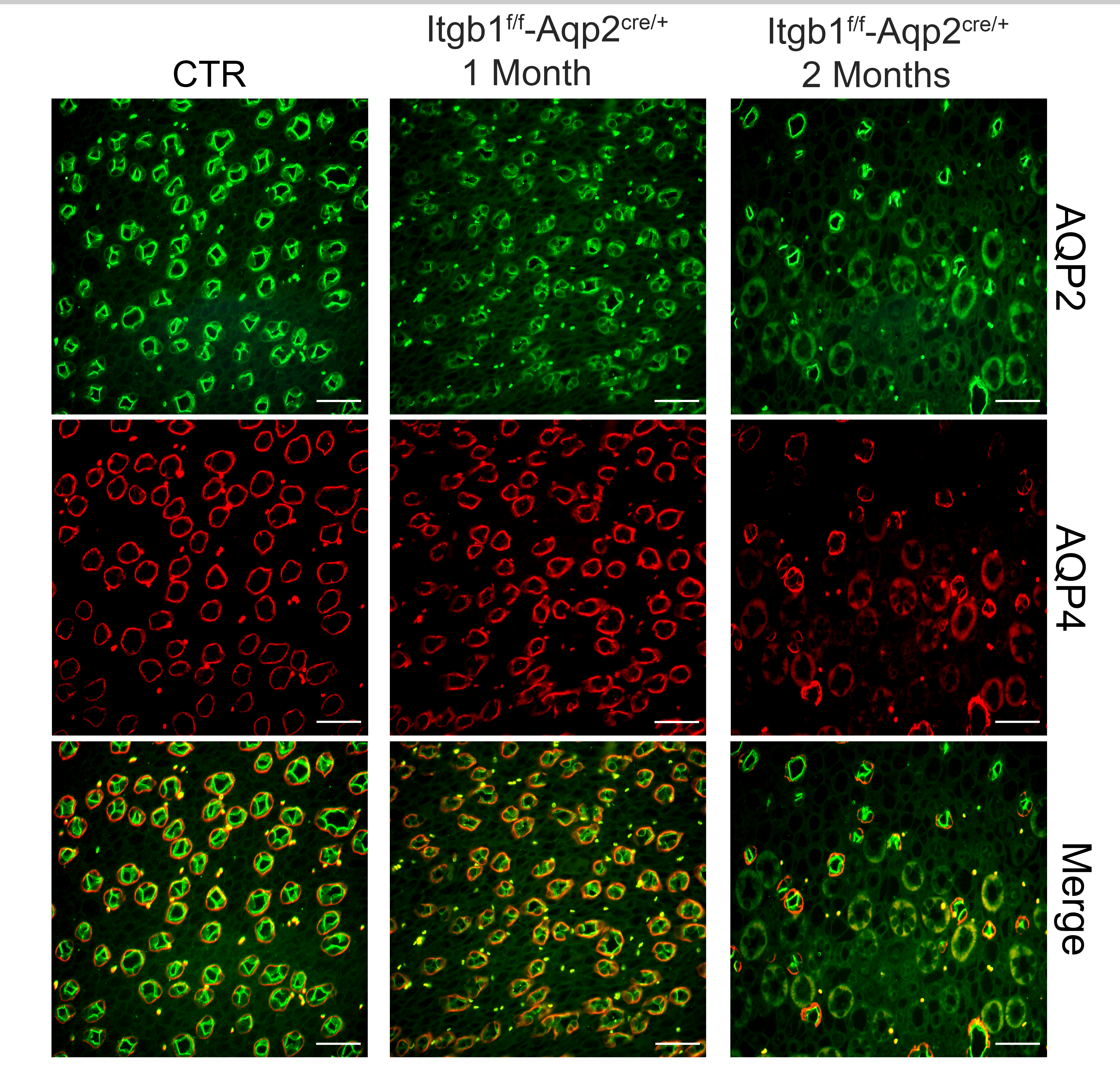

Supplement: Supplementary file 5 [file Image_3.TIF]

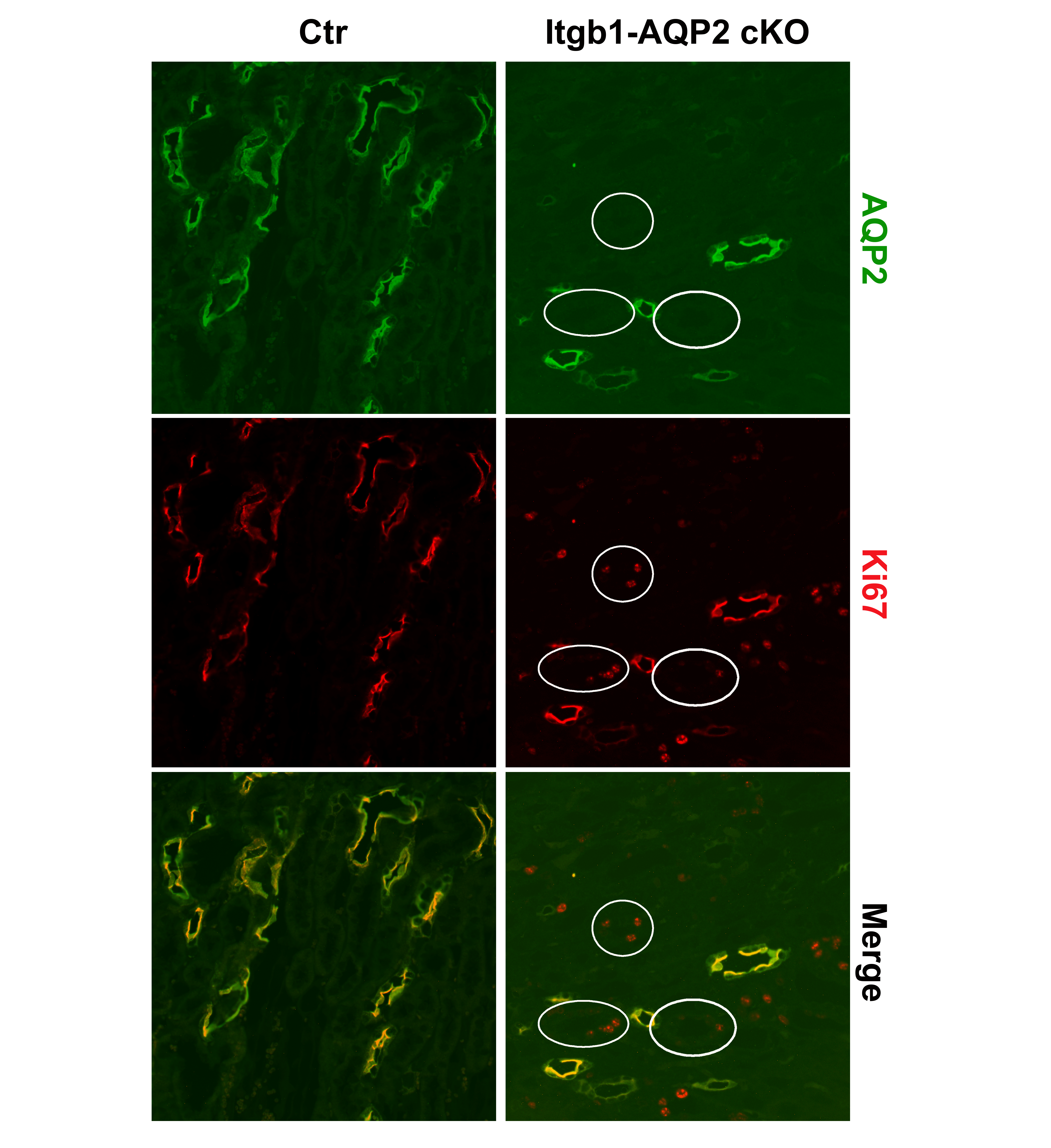

Supplement: Supplementary file 6 [file Image_4.TIF]

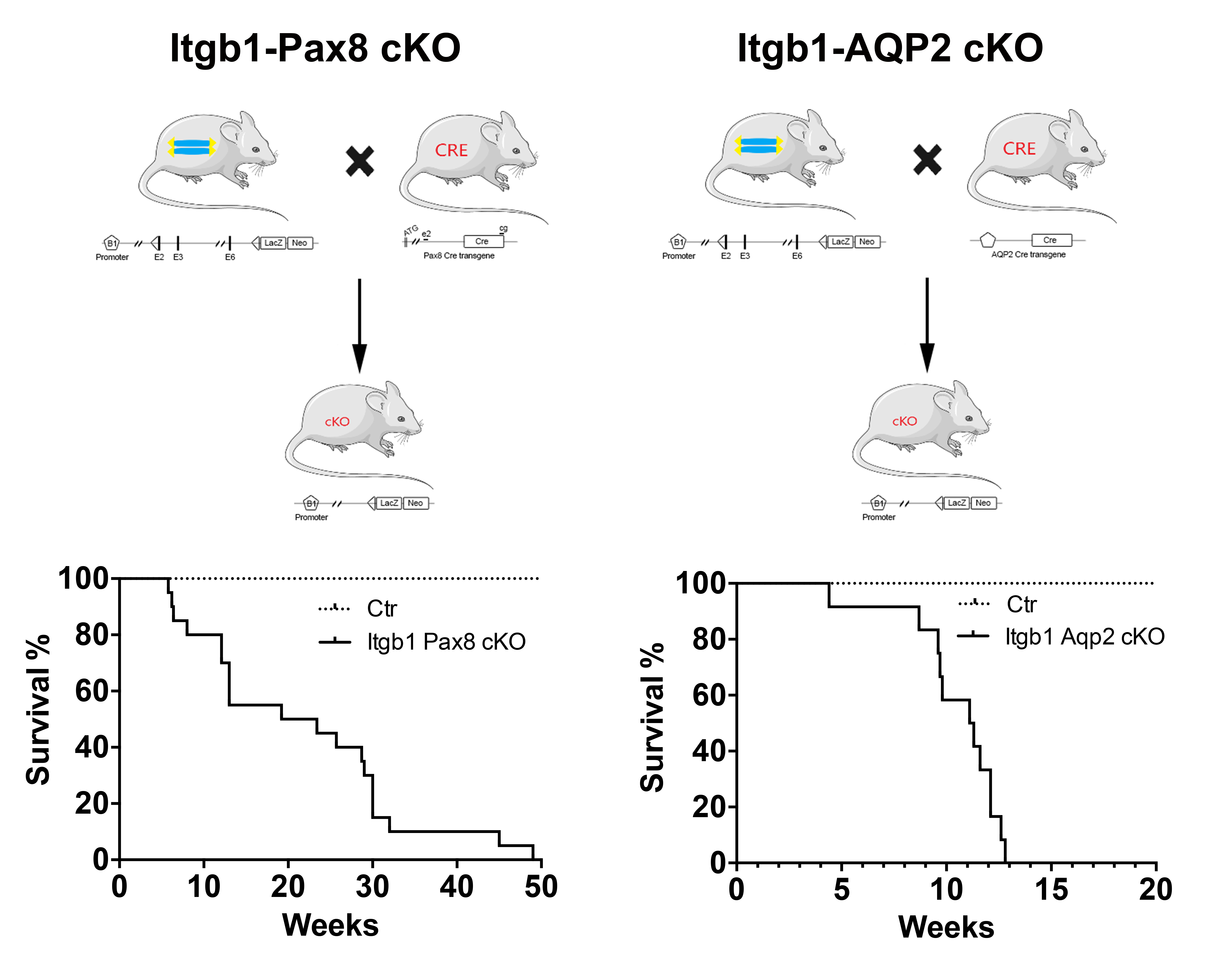

Supplement: Supplementary file 7 [file Image_5.TIF]
